# A Deeper Insight into the Interfacial Behavior and Structural Properties of Mixed DPPC/POPC Monolayers: Implications for Respiratory Health

**DOI:** 10.3390/membranes13010033

**Published:** 2022-12-28

**Authors:** Yingxue Geng, Yan Cao, Yingjie Li, Qun Zhao, Dan Liu, Ge Fan, Senlin Tian

**Affiliations:** 1 Faculty of Environmental Science and Engineering, Kunming University of Science and Technology, Kunming 650500, China; 2 Faculty of Civil and Hydraulic Engineering, Xichang University, Xichang 615013, China

**Keywords:** lipid monolayers, pulmonary surfactant, Langmuir monolayers, phase behavior, intermolecular interaction

## Abstract

1,2-dipalmitoyl-sn-glycero-3-phosphocholine (DPPC) and 1-palmitoyl-2-oleyl-sn-glycerol-3-phosphorcholine (POPC) are important components in pulmonary surfactants (PSs), of which the relative content is related to lung compliance. Herein, the phase behavior and thermodynamic structure of mixed DPPC/POPC monolayers were studied to elucidate the intermolecular interaction between DPPC and POPC molecules. Surface pressure–molecular area isotherms demonstrated that POPC significantly affected the phase behavior of the lipid domain structure as a function of its concentration. The compression modulus of the mixed monolayers reduced with the increase in POPC proportion, which can be attributed to the intermolecular repulsion between DPPC and POPC. Brewster angle microscopy analysis showed that the ordered structure of the monolayers trended toward fluidization in the presence of POPC. Raman spectroscopy results revealed that the change in C–C skeleton stretching vibration was the main cause of the decrease in the monolayer packing density. These findings provide new insights into the role of different phospholipid components in the function of PS film at a molecular level, which can help us to understand the synergy effects of the proportional relationship between DPPC and POPC on the formation and progression of lung disease and provide some references for the synthesis of lung surfactants.

## 1. Introduction

Mixed lipid monolayers are interesting in biophysical processes because complex lipid films occur on such biological interfaces as pulmonary surfactants, gastric mucosa, and the skin [1,2]. In this context, pulmonary surfactants (PSs) are especially important because of their crucial role during respiration. They can effectively reduce the surface tension of alveoli, avoiding alveolar collapse at the end of expiration and facilitating the work of breathing [3]. PSs have a complex structure, composed of 90% lipids and 10% proteins [4]. Among the lipids that constitute PSs, the most abundant (70–80%) components are phosphatidylcholines (PCs), which are mainly responsible for surface activity [5,6]. Approximately half of PCs are molecules with both chains saturated, e.g., 1,2-dipalmitoyl-sn-glycero-3-phosphocholine (DPPC) [7]. DPPC is the only component that can form a liquid condensation (LC) phase at physiological temperature. It is generally accepted that DPPC is the most important substance to reduce alveolar surface tension [8]. Besides DPPC, a non-negligible fraction of PCs in the PSs are unsaturated lipids, which occupy up to 50% of all PCs and consist of mainly PC 16:0–16:1, 16:0–18:2, and 16:0–18:1 [6]. These unsaturated components play a critical role in the spontaneous spreading of PS monolayers at the air–water exchange interface, which is very important for many events that involve phospholipid assembly [9]. Two-component lipid monolayers are another step closer to more complex and hence realistic PS models. Binary mixtures of saturated and unsaturated lipids have been studied for a long time. Some of those works focused on the role of the phosphatidylglycerol (PG) headgroup [10,11,12]. Note, however, that PGs, in contrast to PCs, are only a minor unsaturated lipids class in PSs [6], and thus cannot accurately represent the saturation–unsaturation nature of surfactants in lungs. Therefore, the DPPC/POPC mixture seems to better represent the saturation–unsaturation nature of PSs [7].

1-palmitoyl-2-oleyl-sn-glycero-3-phosphocholine (POPC) is the natural constituent of PS and plays a functional role in the membrane-related respiratory process [13,14]. Previous studies have shown that the relative content of DPPC and POPC changes with the development and physiological state of the human body. In clinical medicine, when patients had lung disease, the percentage of palmitic acid in PCs was significantly decreased, whereas the relative amounts of unsaturated species in PCs increased significantly in all groups [15]. The percentage of DPPC and the ratio of DPPC to POPC in the group of infants who developed respiratory distress syndrome, with or without subsequent chronic lung disease, was significantly lower overall than among the infants who did not develop respiratory distress syndrome [16,17]. These results indicate that the ratio of DPPC to POPC is related to lung compliance. The two components have their own efficacy, and they did not appear one by one, but mixed together, and took different responsibilities in the process of PS monolayer formation. Although the chemical composition of the mixed system is considerably simpler than a natural PS, DPPC/POPC monolayers as the model system have been experimentally proven to simulate certain biophysical properties of the natural PS, such as dynamic surface activity, biomechanics, and phospholipid phase separation upon film compression. Hence, the two-component lipid mixture (the mixed DPPC/POPC monolayers) can indeed be physiologically relevant. Binary mixtures of phospholipids have been studied a lot, and it is known that saturated and unsaturated lipids have an important influence on lung function [10,11]. However, the different roles that DPPC and POPC play in the interfacial activities of PS and the interaction mechanism between the two molecules are not fully understood.

The interfacial behavior and structural properties of mixed DPPC/POPC monolayers were demonstrated in in vitro studies using a wide variety of surface-sensitive techniques. First, a Langmuir–Wilhelmy (L–W) film balance was employed to simulate respiratory and circulatory processes in vitro to investigate the phase behavior of mixed DPPC/POPC monolayers formed by different mol ratios. We found that compared with pure DPPC monolayers, π-A isotherms of mixed DPPC/POPC monolayers shifted toward higher molecular areas, and the values of *C*_S_^−1^ reduced. Next, excess areas and the virial state equation were applied to characterize the interaction between DPPC and POPC molecules, and the results indicate that the dominant interaction between DPPC and POPC molecules is mutual repulsion. Brewster angle microscopy (BAM) analysis further showed that the ordered structure of the monolayers was destroyed in the presence of POPC. Raman spectroscopy revealed that the change in C–C skeleton stretching vibration was the main cause of the decrease in the monolayers’ packing density. The primary objective of this study is to elucidate the phase behavior and thermodynamic structure of the mixed DPPC/POPC monolayers at a molecular level, which can help us to understand the synergic effects of the proportional relationship between DPPC and POPC on the formation and progression of lung disease. To the best of our knowledge, this study is the first to examine the different roles that DPPC and POPC play on the interfacial activities of PS film, which are of relevance to lung compliance, and systematically probe the interaction mechanisms between DPPC and POPC.

## 2. Materials and Methods

### 2.1. Materials

Natural PS was prepared from porcine bronchoalveolar lavage fluid by means of organic solvent extraction, and the composition and purity of the prepared PS were provided in our previous study [18]. The phospholipid spectrum of the prepared PS was similar to that found in Curosurf. DPPC and POPC were purchased as powders with 99% purity from Avanti Polar Lipids (Alabaster, AL, USA) and used without further purification (the chemical structures of these compounds are shown in Figure S1). The organic reagents used in the experiment were certified for analytical grade and were obtained from Kelong Chemical Reagent Co. Ltd. (Chengdu, China). Milli-Q purified water with a resistivity of 18.25 MΩ·cm formulated 0.9% NaCl solution (saline solution) was used as the subphase for all experiments.

### 2.2. Langmuir Film Experiments

Langmuir film experiments were performed on a commercial L-W film balance (JML04C2, Zhongchen Digital Technology Equipment Co., Ltd., Shanghai, China), which was made from polytetrafluoroethylene (PTFE) shaped into a rectangular trough (300 × 100 mm). The system was equipped with an ultra-sensitive surface pressure sensor and two symmetrically moving barriers were used for the compression of the air–water interface. The measuring range of surface tension was 0–150 mN·m^−1^, the resolution was 0.05 mN·m^−1^, and the surface tension was measured using the Wilhelmy plate method. DPPC and POPC mixtures at four different molar ratios (*X*_POPC_ = 0, 0.2, 0.4, 0.6, 0.8 1.0) with a concentration of 1.0 mM were prepared by dissolving phospholipids (or phospholipid mixtures) in chloroform. The appropriate volumes of solution in chloroform were spread onto the air–water interface by dropwise addition using a Hamilton microsyringe. We waited for 20 min to ensure that the chloroform was completely volatilized, and then the compression was initiated with the barriers’ symmetrical movement at a constant rate of 10 mm·min^−1^. π-A isotherms were recorded in the form of surface pressure (mN·m^−1^) against mean area per lipid (APL, Å^2^·molecule^−1^). In all experiments, the temperature of the subphase was kept constant at 37 ± 0.5 °C by a circulating water system. All experiments were repeated at least three times.

### 2.3. Calculation of Compressional Modulus

The compression modulus *C*_S_^−1^ was calculated from π-A isotherms using Equation (1) [19,20]. A and π represent APL and the surface pressure, respectively.

*C*_S_^−1^ = −A(*d_π_*/*d_A_*), (1)

### 2.4. Calculation of Excess Areas and Intermolecular Force

According to the theory of physical chemistry, the interaction between two components can be expressed by the equation of the mixture. When the two components are perfectly mixed, the molecular area of the ideal mixed monolayer *A*_12, ideal_ can be calculated by Equation (2) [21]. Under experimental conditions, the interaction between different molecules is different. Therefore, when the two components interact, the experimental value (*A*_12, exp_) of the average molecular area of the mixed monolayers is different from the theoretical value (*A*_12, ideal_). This difference is called excess molecular area (*A*_12, exp_), which can reflect the interaction between different molecules. *A*_exc_ was calculated from Equation (3) [22].

*A*_12, ideal_= (*A*_1_)_π_*X*_1_ + (*A*_2_) _π_*X*_2_, (2)

*A*_exc_ = *A*_12, exp_ − *A*_12, ideal_, (3)

where *A*_1_ and *A*_2_ are the molecular areas of components 1 and 2 at the same surface pressure, and *X*_1_ and *X*_2_ are the mole fractions of each component in the mixed monolayers. *A*_12, ideal_ is the average molecular area of ideally mixed monolayers. *A*_12, exp_ is the experimental value of the average molecular area in the mixed monolayers under constant surface pressure. For binary mixed monolayers, if the two components undergo ideal mixing or phase separation, the *A*_exc_ will be zero. Deviations from a zero value are taken to indicate miscibility and nonideal mixing, and various types of interactions occur in the film. According to previous reports, the positive and negative deviations represent intermolecular repulsion and attraction, respectively [22]. In addition, the positive and negative deviations from the additivity rule indicate mixing but with unfavorable interactions [23,24].

### 2.5. Virial State Equation

In this study, the virial state equation, in Equations (4) and (5), was applied to characterize the interaction between DPPC and POPC by virial coefficients [22,25].

πA/kT = b_0_ + b_1_π + b_2_π^2^, (4)

(b_1_)_m_ = (b_1_)_1_ X_1_^2^ + (b_1_)_2_ X_2_^2^ + 2(b_1_)_12_ X_1_X_2_ (5)

where *b*_0_, *b*_1,_ and *b*_2_ are virial coefficients. *X* refers to the molar fraction, (*b*_1_)_1_ and (*b*_1_)_2_ are the second virial coefficients of the pure components 1 (DPPC) and 2 (POPC), respectively, and (*b*_1_)_12_ is the second virial coefficient due to interactions between both components. The value of *b*_0_ is attributed to the aggregation state of the film-forming molecules. The value of *b*_1_ provides information on the intermolecular interaction, and negative and positive values represent attraction and repulsion, respectively. The remaining virial coefficients are not significant.

### 2.6. BAM Experiments

The morphology of the mixed DPPC/POPC monolayers was observed by BAM (Nanofilm-EP4 BAM, Accurion GmbH, Göttingen, Germany). A Teflon tray was previously filled with saline solution and placed on the antivibration table. An appropriate volume of lipid mixture was spread on the air–water interface over the subphase solution and was left for 20 min for chloroform evaporation, then the micromorphology of the monolayers at a constant pressure (π = 30 mN·m^−1^) was characterized by BAM. The spreading of the monolayers on the subphase and the acquisition of images acquired were performed at room temperature.

### 2.7. Raman Spectroscopy

The in situ Raman spectroscopy of the mixed DPPC/POPC monolayers was obtained by a laser confocal micro-Raman spectrometer (DXRxi, ThermoScientific, Waltham, MA, USA). The Raman spectrometer was equipped with a sample cell with a diameter of 2 cm. Before the measurement, in order to control the surface pressure of phospholipid monolayers, the amount of lipid solution corresponding to the surface pressure of 30 mN·m^−1^ was measured by a surface tension meter. Then, the suitable amount of the DPPC/POPC (*X*_POPC_ = 0, 0.4, 0.6, 1.0) mixture liquid was spread on the air–water interface. After the volatilization of chloroform, the conformational changes of phospholipids at a surface pressure of 30 mN·m^−1^ were detected. The excitation wavelength of the laser was 633 nm, the laser power was 6.8 mW, the exposure time was 0.00833 s, the number of scans was 900, and the confocal pinhole mode was 50 μM.

## 3. Results and Discussion

### 3.1. The Phase Behavior and Compressibility of the Mixed DPPC/POPC Monolayers

**Figure 1:**
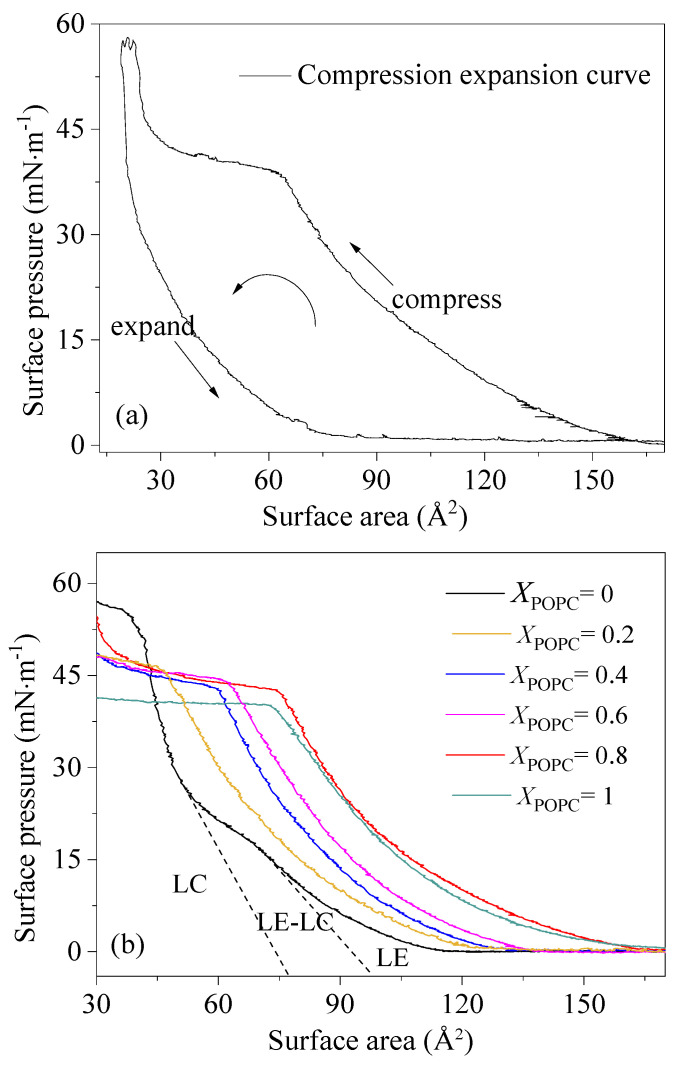
π-A isotherms of porcine PS film (**a**) and mixed DPPC/POPC monolayers (**b**).

The quasi-equilibrium compression of the monolayers provides the π-A isotherms, which is an important characteristic related to the functionality of PS and gives information about the phase behavior and a straightforward characterization of the physicochemical properties of phospholipid monolayers [26,27]. The π-A isotherms for the investigated porcine PS and the mixed systems of DPPC/POPC (*X*_POPC_ = 0, 0.2, 0.4, 0.6, 0.8, 1.0) are presented in Figure 1.

Figure 1 shows the π-A isotherms of porcine PS, DPPC, and the DPPC/POPC mixed phospholipid system (*X*_POPC_ = 0, 0.2, 0.4, 0.6, 0.8, 1.0). As shown in Figure 1a, the surfactant film of porcine PS undergoes regular gas (G), liquid expansion (LE), coexistence of LE and liquid condensation (LC), and LC phases during compression expansion. The compression expansion curve is separated with a large hysteresis area. Similarly, at lower surface pressures, DPPC monolayers were in a disordered LE state, and with the surface pressure increasing, the monolayers adopted an LC state at APL = 0.51 nm^2^, which is consistent with previous reports (Figure S1 and Figure 1b) [28,29,30,31]. The surface pressure of POPC monolayers was systematically above that of DPPC monolayers at a low surface pressure. The isotherms of DPPC/POPC mixed systems appeared between the isotherms of the mono-component systems, especially at a low surface pressure, and as the ratio of POPC increased, the isotherms shifted toward larger molecular areas. For pure POPC monolayers, the surface pressure started to increase when the area decreased to APL = 1.67 nm^2^. With decreasing surface area, the slope of the isotherm increased (i.e., the isotherm is concave upwards). However, when the surface pressure reached 39 mN·m^−1^, the slope of the isotherm decreased with no remarkable increase in the surface pressure, and a phase change plateau appeared. The POPC monolayers can be compressed up to a lateral pressure of 41 mN·m^−1^, whereas the LC phase cannot occur. Corresponding to DPPC, POPC molecules in the liquid crystalline phase were loosely distributed and could result not only in the higher fluidity of the lipid molecules but also in better dispersibility of the aqueous phase. The particle size and dispersion of mixed DPPC/POPC liposomes in pure water were measured. As shown in Table S1, with the increase in POPC content, the average particle size of the micelle decreased and the dispersion index (PDI) decreased, which was in the order of 0.445 > 0.313 > 0.156. The results indicate that POPC has better dispersibility than DPPC in the aqueous phase. The higher fluidity and the better dispersibility of POPC were attributed to the stress curvature induced by the unsaturated acyl chain of POPC, because the presence of a double bond in the acyl chain induces a kink that hampers the tight lipid packing required to sustain high compressions. At a high surface pressure, POPC molecules may be transferred to the aqueous phase and the surface adsorption of POPC molecules is reduced. Thus, the unsaturation in the oleoyl chain was the main reason for the collapse of the monolayer and no LC phase occurrence. The APL corresponding to the plateau was within a range of 0.72–0.36 nm^2^. The figure 0.72 nm^2^ corresponded to the starting point of the plateau, and 0.36 nm^2^ corresponded to the minimum cross-section of the tow chains. Unsaturated components have stronger fluidity and better dispersibility than double-saturated phospholipids, which will enhance the overall fluidity of the PS system. At the same time, the presence of unsaturated components also limits other behaviors of bisaturated components. If the concentration of unsaturated phospholipids is high, the integrity of the PS film system will become worse.

**Figure 2:**
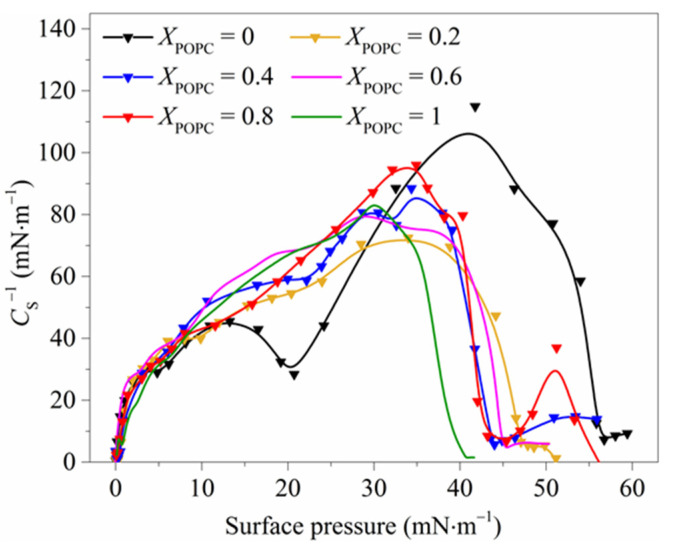
The compression modulus (*C*_S_^−1^) of mixed DPPC/POPC monolayers.

The compression modulus *C*_S_^−1^ is an important parameter characterizing the physical state of the monolayers, reflecting the monolayers’ deformation resistance. The higher the value, the stronger the rigidity of the monolayers [32]. As shown in Figure 2, the values of *C*_S_^−1^ for pure DPPC monolayers were more than 110 mN·m^−1^ within the surface pressure range of 35–45 mN·m^−1^, suggesting that the monolayers were in the LC phase. The value of *C*_S_^−1^ of mixed DPPC/POPC monolayers was lower than that of pure DPPC monolayers, and the *C*_S_^−1^_max_ value of the pure DPPC monolayers was 115 mN·m^−1^ (π = 41.76 mN·m^−1^), nearly 1.5 times larger than that of the mixed monolayers, indicating that DPPC monolayers were more ordered and rigid. This result is in good agreement with the reduction in the maximum surface pressure (*π*_max_), as shown in Figure S2, which was mainly caused by molecular rearrangement processes in the monolayer starting in the coexisting region. These results indicate that the interfacial stability of the mixed monolayers was weakened in the presence of POPC, and hence POPC seems to make the response of the monolayers to lateral compression milder [7].

### 3.2. Homogeneous Analysis of the Mixed DPPC/POPC Monolayers

**Figure 3:**
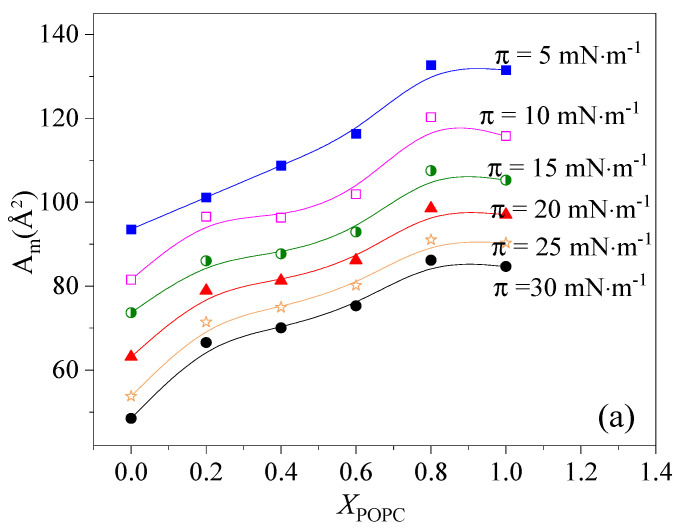

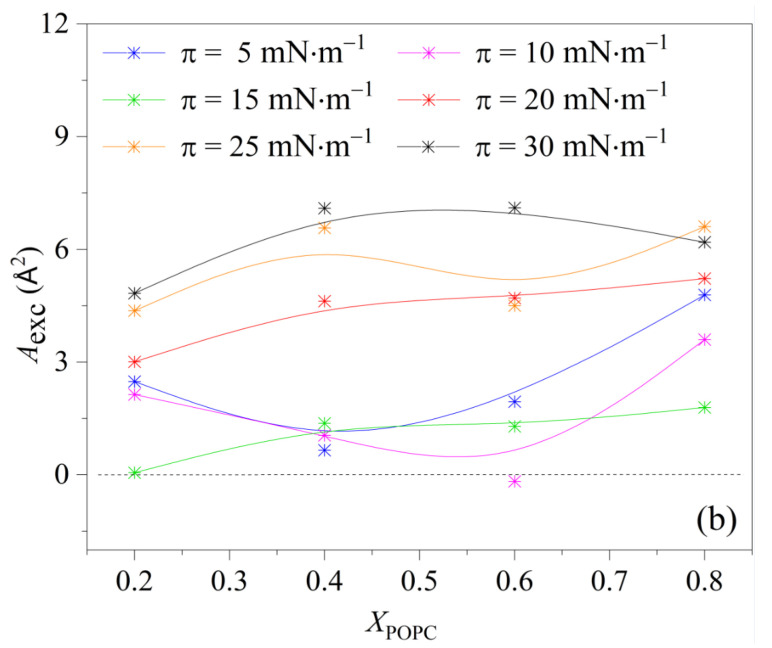
Area per molecule (**a**) and excess area (*A*_exc_) (**b**) in the film versus POPC molar fraction (*X*_POPC_) in mixed DPPC/POPC monolayers.

To analyze the magnitude of condensation and the interaction between DPPC and POPC, the values of *A*_exc_ were calculated according to Equations (2) and (3). These results are presented as a function of POPC proportion in Figure 3. The results show that the dominant interaction between DPPC and POPC molecules is mutual repulsion and with the increase in the lateral surface pressure, the repulsion force gradually increases. Less favorable interactions between DPPC and POPC molecules can lead to partial phase segregation. Notably, when 0.4 < *X*_POPC_ < 0.6, the values of |*A*_exc_| approached zero at low surface pressure (<15 mN·m^−1^), indicating that the two components mix ideally at low surface pressure (<15 mN·m^−1^) [24,33]. It was concluded that the greatest condensation of the mixed monolayers can be induced by changing the relative content of POPC and DPPC, and the thermodynamically favorable binary monolayers of a defined stoichiometry can be formed.

### 3.3. The Intermolecular Interaction between DPPC and POPC Molecules

The Langmuir film is not a thermodynamic stable state but can be treated as a metastable state. Reports in the literature have shown that gaseous monolayers are satisfactorily described by Volmer’s equation [34]. The two-dimensional equation of state for the gaseous monolayer can also be extended to further monolayer stages in which an additional condensed phase is formed owing to two-dimensional aggregation. The two-dimensional virial equation of state (2D-VES) which was deducted by Volmer’s equation was commonly employed to describe the π-A isotherms of the insoluble monolayers in the compressing process including LE and LC states [35,36]. This equation is applicable not only for pure component monolayers but also for binary mixture monolayers. Here, the two-dimensional virial expansion was used to describe deviations from the ideal behavior of the mixed DPPC/POPC monolayers. The nonideality is captured in the second and third virial coefficients which provide information on the lateral headgroup interactions. The π-A isotherms of DPPC/POPC monolayers were further analyzed by Equations (4) and (5). These equations describe the fluidlike state of the monolayer at *A* > *A*c (*A*c: the value of area per lipid molecule at the beginning of the two-dimensional phase transition point) where small 2D aggregates can also occur. The fitting curves of πA/kT vs. π for the several studied compositions are shown in Figure S3, which can be adjusted with a polynomial of 2n degree. The virial coefficients (*b*_0_, *b*_1_, *b*_2_), obtained from Equations (4), and the values of (*b*_1_)_12_, obtained from Equation (5), are listed in Table 1.

The value of *b*_0_ is lower than 1, indicating that two-dimensional phospholipid aggregates may be formed under low surface pressure. The *b*_0_ value of the POPC monolayer is higher than that of DPPC, and *b*_1_ is positive, indicating that the main interaction between phospholipid molecules is repulsive [37], which is consistent with the result of *A*_exc_. The value of (*b*_1_)_12_ (*X*_POPC_ = 0.4) is 0.2274 and is the lowest, significantly, while the corresponding value of *A*_exc_ is close to 0 at a low surface pressure. It can be inferred that *X*_POPC_ = 0.4 is a more favorable mixing ratio compared with the other proportions at a low surface pressure. Comparing the values of *b*_1_ for DPPC and POPC, it was observed that POPC presents the largest value. As *b*_1_ is related to exclusion volumes and interactions between molecules, the double bond of POPC provides higher exclusion volumes and repulsive interactions between molecules [22], which may be the main reason for the reduction in the interfacial stability of the mixed system.

### 3.4. Micromorphology of the Mixed DPPC/POPC Monolayers

To gain complementary information about the lateral organization of the lipid monolayers at the air–water interface, BAM was employed. During the respiratory cycle, the PS monolayers in the alveoli were subjected to a periodic area perturbation of about 30–40%, with a reference state characterized by a value of 30 mN·m^−1^ [27,38]. In this experiment, the micrograph of the floating monolayers was characterized by BAM at a constant pressure (π = 30 mN·m^−1^).

**Figure 4:**
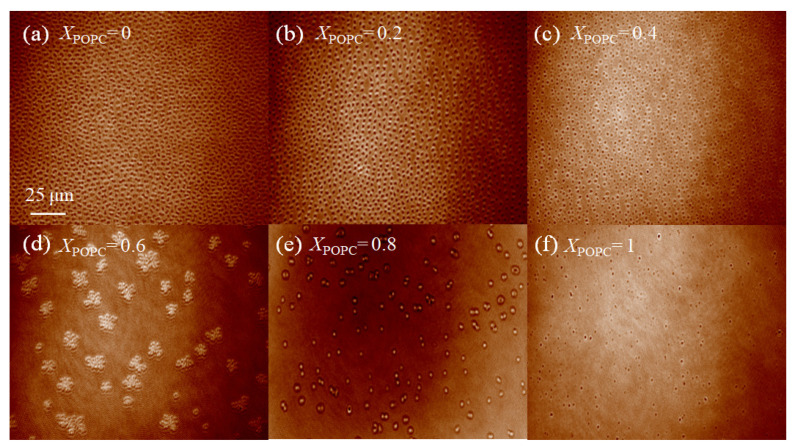
BAM micrographs (490 × 610 μm^2^) of mixed DPPC/POPC monolayers at air–water interface at the constant surface pressure (π) of 30 mN·m^−1^. (**a**) DPPC, (**b**) DPPC/POPC (*X*_POPC_ = 0.2), (**c**) DPPC/POPC (*X*_POPC_ = 0.4), (**d**) DPPC/POPC (*X*_POPC_ = 0.6), (**e**) DPPC/POPC (*X*_POPC_ = 0.8), (**f**) POPC.

As shown in Figure S4, DPPC monolayers initially experienced a phase change from LE to LC at a low surface pressure of 8 mN·m^−1^. When the monolayers were compressed, the size of LC domains increased. When the surface pressure was 30 mN·m^−1^, DPPC monolayers were in the ordered LC phase, as shown in Figure 4a. When *X*_POPC_ = 0.2, the mixed monolayers existed in the LC phase (Figure 4b). When *X*_POPC_ = 0.4, mixed monolayers were dispersed homogeneously and existed in the LE phase (Figure 4c). However, when *X*_POPC_ was increased to 0.6 and 0.8, the monolayers had a phase separation structure. The monolayer forms more small-area structures of separate LC domains (Figure 4d,e). Figure 4d shows that the mixed DPPC/POPC monolayers presented in multilobed domains. This may be the result of domains that nucleate in close proximity and form a bridge that persists through compression [39]. Unsaturated POPC monolayers exist as a single homogenous LE (fluid) phase without any clear indication of a transition from LE to LC, even at higher surface pressures (Figure 4f). The unsaturated POPC monolayers have a lower bulk density and larger molecular areas than those of saturated DPPC monolayers at the physiologically relevant surface pressure. This is consistent with the literature reports [40]. From these observations, it can be inferred that POPC can increase the liquidity of the mixed monolayers and destroy the packing status of DPPC molecules.

### 3.5. The Structure and Conformation of Phospholipid Molecules

**Figure 5:**
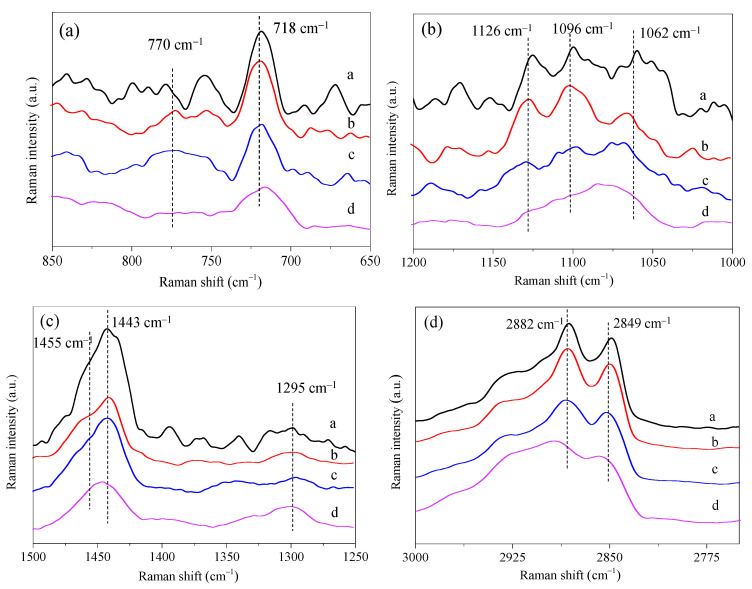
Raman spectra of mixed DPPC/POPC monolayers ((**a**): DPPC, (**b**): DPPC/POPC (*X*_POPC_ = 0.4), (**c**): DPPC/POPC (*X*_POPC_ = 0.6), (**d**): POPC) at the air–water interface.

The intermolecular repulsion force between DPPC and POPC molecules is responsible for the inhibitive effect of POPC on the interfacial stability of PS film. However, the contributions of molecular conformation to the structural properties of the mixed monolayers were not determined. Raman spectra of the mixed DPPC/POPC monolayers at the air–water interface are shown in Figure S5. The absorption at 718 cm^−1^ corresponds to the C−N stretching vibration of the headgroup of PCs [41,42]. That at 770 cm^−1^ is assigned to C−N trans stretching vibrations [42]. As shown in Figure 5a, when DPPC and POPC were mixed at different proportions, the absorption at 718 cm^−1^ remained unchanged, and there was no obvious vibration peak at 770 cm^−1^, indicating that the polar head was parallel to the surface of the monolayers. The stretching vibration of the C–C skeleton in the range of 1000–1200 cm^−1^ can be used to characterize the trans/gauche conformation changes of the phospholipid alkyl chain. The in-plane and out-of-plane C–C stretching vibration mainly shows three peaks of 1062, 1096, and 1126 cm^−1^ (Figure 5b). The vibration at 1062 cm^−1^ and 1096 cm^−1^ was attributed to the stretching vibration of the C–C bond for the trans and gauche conformations, respectively [43].

The ratio of the intensity of the C–C trans symmetric stretching mode (1062 cm^−1^) and C–C trans asymmetric stretching mode (1126 cm^−1^) to the C–C gauche conformer stretching (1096 cm^−1^) is indicative of acyl-chain ordering [44]. As shown in Table 2, the peak intensity ratios *I*_1062_/*I*_1096_ and *I*_1126_/*I*_1096_ gradually decreased with the increase in the content of POPC in the mixed monolayers. The results indicate that the vibration of the gauche stretching mode of C–C bonds was enhanced, and the fluidity of the mixed monolayers increased compared with DPPC monolayers. The stretching vibration of methylene C–H bond occurs in the range of 2750–3000 cm^−1^ (as shown in Figure 5d), while 2849 and 2882 cm^−1^ are symmetric and antisymmetric stretching vibrations of methylene groups in DPPC molecules, respectively. They are particularly sensitive to the conformation of DPPC aliphatic chains. As reported in the literature, the peak ratio *I*_2882_/*I*_2849_ reflects the acyl-chain lateral packing density and more specifically, these ratios can be used as an index of the change in the proportion between disorder and order in the conformation of the alkyl chain [45]. As shown in Table 2, the peak ratio *I*_2882_/*I*_2849_ decreased slightly when *X*_POPC_ = 0.4, showing that the conformation of methylene C–H bond changed, and the ordered arrangement of acyl chains is reduced. *I*_1443_/*I*_1455_ represents the lattice structure of acyl chains (Figure 5c) [44], and the ratio of *I*_1443_/*I*_1455_ is in the order of DPPC > DPPC/POPC (*X*_POPC_ = 0.4) > DPPC/POPC (*X*_POPC_ = 0.6) > POPC, suggesting that the order degree of the mixed monolayers reduced. This result is consistent with the increase in the gauche stretching mode of C–C bonds, which can enhance the disorder degree of acyl chains.

Huynh et al. pointed out that the two saturated acyl chains of DPPC can be compressed and arranged in a straight line [40], while in the case of the POPC molecule, only one hydrocarbon tail is saturated so that the acyl chains of POPC are curved, which makes it not as densely packed as the DPPC molecules are [20,46]. This result emphasizes the effect of acyl chains on the interface stacking structure of phospholipid molecules, but the interaction mechanism is not elucidated. Although DPPC and POPC have similar molecular volumes, the area occupied per lipid headgroup of POPC at room temperature is larger than that for DPPC [47]. With regard to these results, we hypothesized that the stability of phospholipid monolayers is due to the gauche stretching mode of C–C bonds being enhanced. In addition to the effect of acyl chains, the headgroup’s interaction with DPPC and POPC can also influence the stability of phospholipid monolayers. With the lateral pressure increasing, the APL is reduced, and the headgroup P^−^–N^+^ of phospholipid molecules can be converted from horizontal to vertical [7]. The amino group (NH_3_^+^) is positively charged and the phosphoric acid (PO_3_^−^) is negatively charged, and the polar headgroup can be simplified as a P^−^–N^+^ dipole. The hydrophobic chains are mainly bound by the van der Waals force; however, the interaction of the hydrophilic headgroup is mainly manifested as repulsion between headgroup dipoles. The antagonism between the van der Waals force and electrostatic repulsion is a key factor affecting the stability of the mixed monolayers.

## 4. Conclusions

This study systematically assesses the phase behavior and thermodynamic structure characteristics of the mixed DPPC/POPC monolayers at the air–water interface for the first time. The results show that the relative content of DPPC and POPC can significantly affect the phase behavior and micromorphology of the mixed monolayers. With the increase in the POPC ratio, the plateau of the LE-LC phase coexistence became wider, and the LE phase expanded. In addition, the interfacial stability of the mixed monolayers was weakened in the presence of POPC, which was attributed to the intermolecular repulsion force between DPPC and POPC molecules. The in-plane and out-of-plane C−C stretching vibration is a key factor affecting the intermolecular repulsion force, and the enhanced gauche stretching mode of the C−C bond points to the main reason leading to the reduction in the stability of the mixed monolayers. The variation in the phase behavior and the microstructure of the mixed DPPC/POPC monolayers can provide evidence of the inhibitory effect of unsaturated components on the PS film and even explain the adverse effects of an imbalance in lipid content on the compliance of lung ventilation. These findings are of special importance not only for understanding the physicochemical properties of PS film at the molecular level but also for supporting and guiding PS-related drug synthesis.

## Figures and Tables

**Table 1:** Virial coefficients and correlation coefficients from the fitting of the values.

	*X*_POPC_					
	0	0.2	0.4	0.6	0.8	1
*A*_c_ (Å^2^)	63.68	54.56	59.94	62.19	74.93	72.68
*b*_0_	0.0310	0.0696	0.0417	0.0243	0.1138	0.1101
*b*_1_	0.2292	0.2328	0.2372	0.2545	0.2843	0.2847
*b*_2_	−0.0041	−0.0025	−0.0024	−0.0025	−0.0028	−0.0029
(*b*_1_)_12_	/	0.2335	0.2274	0.2403	0.2966	/
*R* ^2^	0.9991	0.9985	0.9989	0.9992	0.9979	0.9992

**Table 2:** Peak intensity ratios (*I*_a_/*I*_b_) corresponding to the mixed DPPC/POPC monolayers.

Sample	*I*_1126/1096_	*I*_1062/1096_	*I*_2882/2849_	*I*_1443/1455_
DPPC	1.00	1.07	1.16	1.22
DPPC/POPC (*X*_POPC_ = 0.4)	0.92	1.04	1.14	1.18
DPPC/POPC (*X*_POPC_ = 0.6)	0.80	1.03	1.15	1.17
POPC	0.77	0.98	1.15	0.99

## Data Availability

Not applicable.

## Supplementary Materials

The following supporting information can be downloaded at: https://www.mdpi.com/article/10.3390/membranes13010033/s1, Figure S1. The structural formula of DPPC and POPC; Figure S2. The hysteresis curves of DPPC and POPC monolayers; Figure S3. Fitting curves of (πA/kT) vs. π for the mixtures of DPPC/POPC. The dot lines represent the experimental data, and the solid lines represent the regression results of Equation (4); Figure S4. BAM images of the investigated DPPC monolayers at different surface pressures; Figure S5. Raman spectra of the mixed DPPC/POPC monolayers at the air-water interface. a: DPPC, b: DPPC/POPC (XPOPC=0.4), c: DPPC/POPC (XPOPC=0.6), d: POPC; Table S1 Particle size and dispersion index (PDI) of the mixed DPPC/POPC liposomes.
