# Supplementary material for: A Deeper Insight into the Interfacial Behavior and Structural Properties of Mixed DPPC/POPC Monolayers: Implications for Respiratory Health
Source: Membranes (Basel). 2022 Dec 28;13(1):33. doi: 10.3390/membranes13010033 (PMC9864691; doi:10.3390/membranes13010033)
Supplement: Supplementary file 1 [file membranes-13-00033-s001.zip › membranes-1853755-supplementary.pdf]

A Deeper Insight into the Interfacial Behavior and Structural Properties of Mixed DPPC/POPC Monolayers: Implications for Respiratory Health

Figure S1 The structural formula of DPPC and POPC.

Figure S2 The hysteresis curves of DPPC and POPC monolayers

Figure S3 Fitting curves of (πA/kT) vs. π for the mixtures of DPPC/POPC.

Figure S4 BAM images of the investigated DPPC monolayers at different surface pressures.

Figure S5 Raman spectra of the mixed DPPC/POPC monolayers at the air-water interface.

Table S1 Particle size and dispersion index (PDI) of the mixed DPPC/POPC liposomes

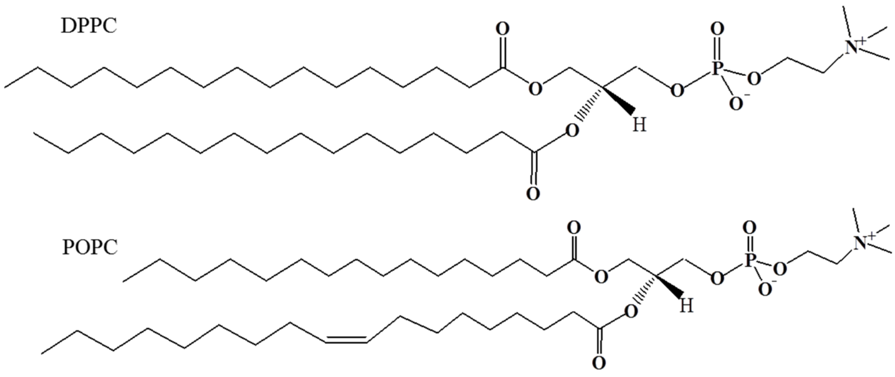

Figure S1. The structural formula of DPPC and POPC.

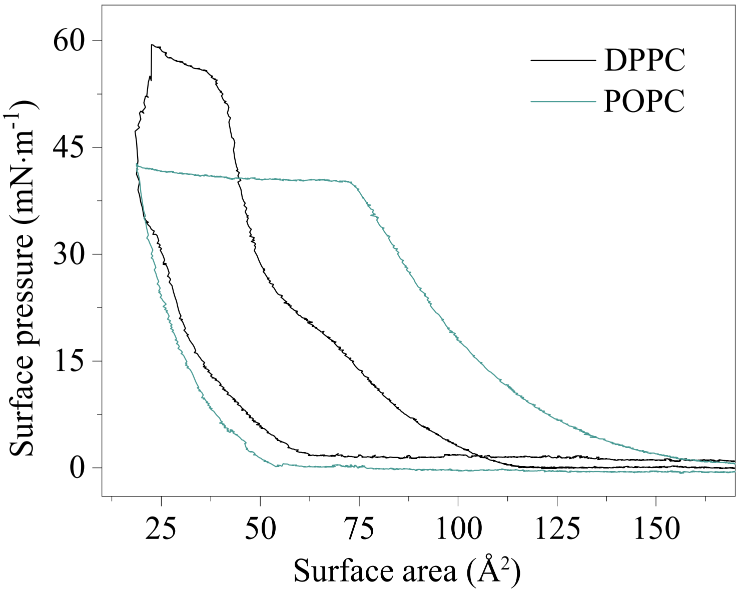

Figure S2. The hysteresis curves of DPPC and POPC monolayers.

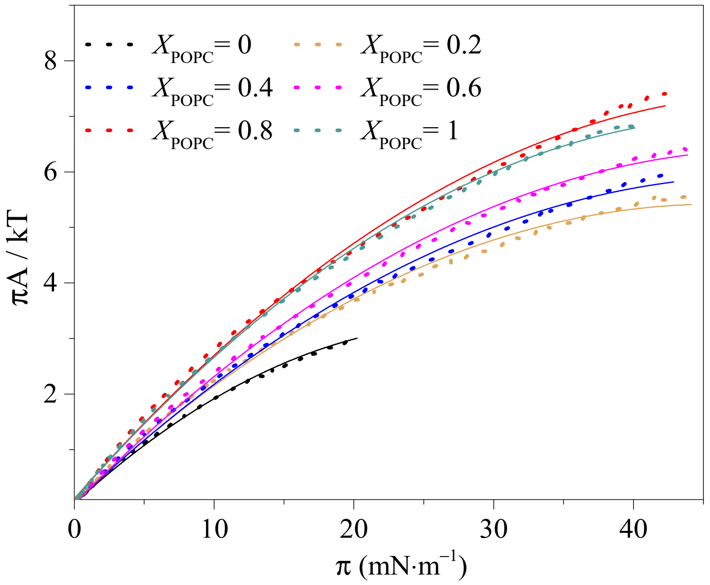

Figure S3. Fitting curves of (πA/kT) vs. π for the mixtures of DPPC/POPC. The dot lines represent the experimental data, and the solid lines represent the regression results of eq (4).

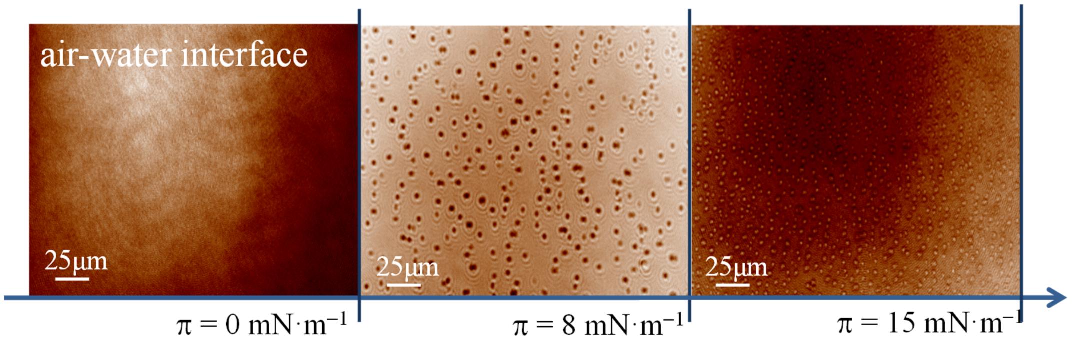

Figure S4. BAM images of the investigated DPPC monolayers at different surface pressures.

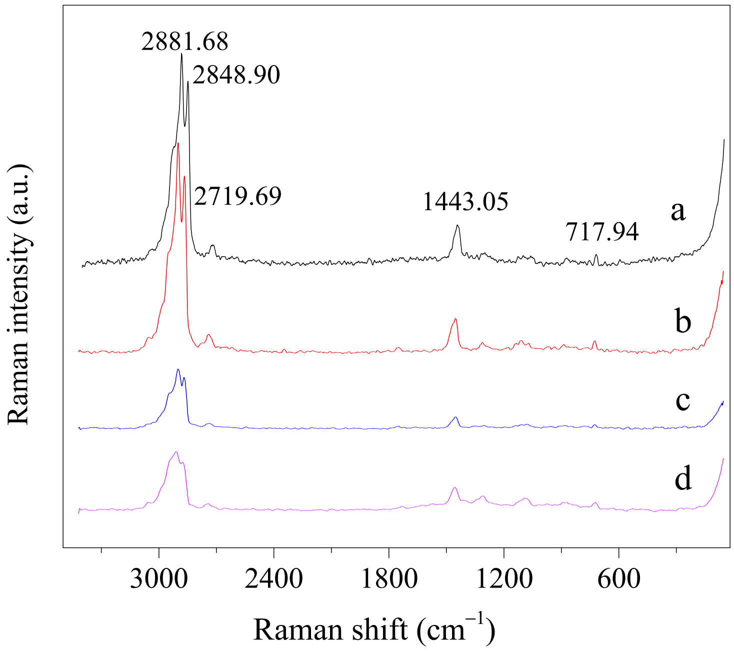

Figure S5. Raman spectra of the mixed DPPC/POPC monolayers at the air-water interface.

a: DPPC, b: DPPC/POPC (X_{POPC}=0.4), c: DPPC/POPC (X_{POPC}=0.6), d: POPC.

Table S1 Particle size and dispersion index (PDI) of the mixed DPPC/POPC liposomes

X_{POPC}	Average particle size (nm)	PDI
0.0	1458	0.445
0.5	1085	0.313
1.0	874	0.156
